# Predictive Value and Diagnostic Potential of IL-10, IL-17A, IL1-β, IL-6, CXCL, and MCP for Severe COVID-19 and COVID-19 Mortality

**DOI:** 10.3390/biomedicines12071532

**Published:** 2024-07-10

**Authors:** Roxana-Elena Cîrjaliu, Ioan-Tiberiu Tofolean, Doina-Ecaterina Tofolean, Anca Chisoi, Cristian Oancea, Emanuela Vastag, Monica Marc, Felix Bratosin, Ovidiu Rosca, Ariadna-Petronela Fildan

**Affiliations:** 1 Faculty of Medicine, “Ovidius” University of Constanta, 900470 Constanta, Romania; roxana.cirjaliu@365.univ-ovidius.ro (R.-E.C.); ioan.tofolean@365.univ-ovidius.ro (I.-T.T.); doina.tofolean@univ-ovidius.ro (D.-E.T.); petronela.fildan@365.univ-ovidius.ro (A.-P.F.); 2 Center for Research and Development of the Morphological and Genetic Studies of Malignant Pathology (CEDMOG), “Ovidius” University of Constanta, 900591 Constanta, Romania; anca.chisoi@365.univ-ovidius.ro; 3 Center for Research and Innovation in Precision Medicine of Respiratory Diseases, “Victor Babes” University of Medicine and Pharmacy Timisoara, 300041 Timisoara, Romania; oancea@umft.ro (C.O.); emanuela.tudorache@umft.ro (E.V.); 4 Discipline of Infectious Diseases, “Victor Babes” University of Medicine and Pharmacy Timisoara, 300041 Timisoara, Romania; felix.bratosin@umft.ro (F.B.); ovidiu.rosca@umft.ro (O.R.)

**Keywords:** COVID-19, SARS-CoV-2, respiratory infections, inflammation

## Abstract

The severe acute respiratory syndrome coronavirus 2 (SARS-CoV-2) necessitates advanced prognostic tools to anticipate disease progression and optimize patient outcomes. This study evaluates the predictive value and diagnostic potential of interleukins interleukin (IL) IL-10, IL-17A, IL1-β, IL-6, chemokine ligand (CXCL), and Monocyte Chemotactic Protein (MCP) for severe coronavirus disease 2019 (COVID-19) and COVID-19 mortality, aiming to correlate cytokine levels with disease severity. Conducted from January 2023 to January 2024, this prospective cohort study involved patients hospitalized with moderate and severe COVID-19 from Romania. This study analyzed statistically significant predictors of severe COVID-19 outcomes. IL-6 and MCP emerged as significant, with hazard ratios (HRs) of 2.35 (95% confidence interval (CI): 1.54–3.59, *p* = 0.014) and 2.05 (95% CI: 1.22–3.45, *p* = 0.007), respectively. Compound scores integrating multiple inflammatory markers also demonstrated predictive value; Compound Score 2 had an HR of 2.23 (95% CI: 1.35–3.68, *p* = 0.002), surpassing most single markers in association with severe disease. Notably, interleukins IL-10 and IL-1β did not show significant associations with disease severity. This study underscores the importance of IL-6 and MCP as robust predictors of severe COVID-19, substantiating their role in clinical assessments to foresee patient deterioration. The utility of compound scores in enhancing predictive accuracy suggests a composite approach may be more effective in clinical settings.

## 1. Introduction

The coronavirus disease 2019 (COVID-19) pandemic, caused by the novel coronavirus severe acute respiratory syndrome coronavirus disease 2 (SARS-CoV-2), has profoundly impacted global health systems and economies since its emergence in late 2019 [1]. As of mid-2024, the virus has infected millions worldwide, with the total number of cases surpassing 750 million and deaths exceeding 7 million [2,3]. The pandemic has displayed a dynamic epidemiology, with fluctuating incidence rates influenced by factors such as viral mutations, public health interventions, and the global rollout of vaccination programs [4,5,6]. 

COVID-19 presents with a spectrum of clinical manifestations ranging from mild, nonspecific symptoms to severe respiratory distress and multiorgan failure, the latter often leading to fatal outcomes [7,8]. Critical complications such as acute respiratory distress syndrome (ARDS), sepsis, thromboembolism, and multiorgan dysfunction are prevalent among severe cases [9,10,11]. Early and accurate diagnosis of COVID-19 has been central to global efforts to control the spread of the virus. Initially, diagnostic approaches focused primarily on molecular testing for the presence of SARS-CoV-2. As the pandemic progressed, the role of serological and inflammatory markers gained prominence, aiding in the assessment of disease severity and progression [12,13]. 

The ability to predict which patients with COVID-19 are at risk of developing severe complications can significantly alter clinical outcomes [14,15]. Early prediction facilitates timely therapeutic interventions, optimizing resource allocation, and improving patient management strategies. Laboratory parameters such as cytokine profiles have emerged as potential predictive biomarkers for severe COVID-19 [16,17]. Cytokines like interleukin (IL) IL-10, IL-17A, and IL-1β play pivotal roles in the inflammatory response associated with severe COVID-19. Elevated levels of these cytokines might correlate with hyperinflammation and an increased risk of severe outcomes, including death [18,19]. 

The current study aims to investigate the predictive value and diagnostic potential of interleukins IL-10, IL-17A, IL-1β, IL-6, CXCL, and MCP for severe COVID-19 and COVID-19 mortality. The primary objective of this study is to assess the predictive value and diagnostic potential of specific interleukins (IL-10, IL-17A, IL-1β, IL-6), chemokines (CXCL), and monocyte chemoattractant protein (MCP) in predicting severe COVID-19 outcomes and mortality. By correlating elevated or reduced levels of these cytokines with the severity of the disease, this study aims to contribute significantly to the development of prognostic tools that can assist clinicians in anticipating disease progression and tailoring treatment strategies to optimize patient outcomes. The secondary objective revolves around validating a compound scoring system that integrates multiple inflammatory markers, aiming to enhance the predictive accuracy for severe COVID-19, thus providing a robust model that could potentially guide clinical decisions and therapeutic interventions.

## 2. Materials and Methods

### 2.1. Study Design

This prospective cohort study was conducted on a database of patients that spanned from January 2023 to January 2024 at the Victor Babes Hospital for Infectious Disease and Pulmonology from Timisoara, in collaboration with the Clinical Hospital for Infectious Disease from Constanta, Romania. This study focused on evaluating the predictive role of laboratory parameters during hospital admission for SARS-CoV-2 infections in determining the risk of severe COVID-19 and mortality risk. Our cohort included all patients with a positive COVID-19 PCR test that required hospitalization.

Ethical approval for this study was granted by the Institutional Review Board of the participating hospitals, ensuring adherence to the ethical standards established by the 1964 Helsinki Declaration and its later amendments, which pertain to human research ethics. Informed consent was obtained from all patients before their inclusion in the study, guaranteeing their understanding and voluntary participation in the research.

Upon admission, each participant had various laboratory parameters measured, including a complete blood count, C-Reactive Protein (CRP), and interleukins IL-10, IL-17A, and IL-1β using ELISA techniques. Other biochemical markers for organ function and damage were also assessed. The study groups were divided based on the severity of COVID-19—moderate and severe—determined by clinical criteria such as oxygen saturation and respiratory rates.

### 2.2. Inclusion and Exclusion Criteria

The inclusion criteria comprised the following: (1) Confirmed Diagnosis: All participants included in this study must have a confirmed diagnosis of SARS-CoV-2, evidenced by a positive result from a Polymerase Chain Reaction (PCR) test; (2) Hospitalization Requirement: Eligible participants are those who required hospitalization for COVID-19; (3) Age Consideration: This study includes participants of all ages, to encompass the wide demographic impact of COVID-19 across different age groups; (4) Consent: Participation in the study is contingent upon individuals providing informed consent, affirming their voluntary involvement and comprehension of the research purposes and procedures; (5) Timing of Hospital Admission: Participants were included if they were admitted to the hospital during the study period from October 2022 to January 2024.

The exclusion criteria comprised the following: (1) Previous Severe COVID-19: Patients who had previously been hospitalized for severe COVID-19 prior to the study period are excluded to avoid the confounding effects of prior severe disease and interventions on current health outcomes; (2) Co-infection: Individuals diagnosed with co-infections at the time of admission, such as influenza or bacterial pneumonia, which could alter the inflammatory response, are excluded to maintain a focus on COVID-19 specific responses; (3) Incomplete Data: Patients lacking complete initial laboratory or clinical data necessary for the assessment of the specified interleukins (IL-10, IL-17A, IL-1β) upon hospital admission are excluded; (4) Withdrawal of Consent: Any patient who withdraws consent at any point during the study is excluded from the final analysis to comply with ethical guidelines; (5) Participation in Other Studies: Patients currently participating in other interventional studies that might interfere with the cytokine measurements or outcomes of interest are excluded to prevent interaction effects. 

### 2.3. Biochemical Analysis

A complete blood count (CBC), including white blood cell (WBC) count, was conducted using a Sysmex XN-550 automated hematology analyzer from Sysmex Corporation, Kobe, Japan. For this analysis, each sample required 1 mL of venous blood collected in a tube containing Ethylenediaminetetraacetic acid (EDTA) as an anticoagulant to prevent clotting and preserve sample integrity. Levels of C-Reactive Protein (CRP) were measured using a Cobas Integra 400 Plus or Cobas e411 analyzer from Roche Diagnostics GmbH, Mannheim, Germany. These assays required 2 mL of blood collected in tubes with a separator gel to facilitate serum separation. In addition to CRP, interleukins IL-10, IL-17A, and IL-1β were quantified using enzyme-linked immunosorbent assay (ELISA) techniques. Other biochemical markers indicative of organ function and cellular damage were analyzed using spectrophotometry-based biochemical analyzers.

The levels of CXCL and MCP were quantified using enzyme-linked immunosorbent assay (ELISA) techniques. Blood samples were collected in tubes containing Ethylenediaminetetraacetic acid (EDTA) to prevent clotting. These samples were then processed to separate the plasma, which was subjected to ELISA specific for CXCL and MCP. This process involved coating microplates with antibodies specific to each chemokine, incubating with the plasma, and then using a secondary enzyme-linked antibody that binds to the complex. A substrate was added to produce a measurable color change, with the intensity reflecting the chemokine concentration, which was quantitatively assessed using a spectrophotometer.

The severity of COVID-19 in this study was classified according to existing guidelines [20]. The admitted patients were either with moderate or severe COVID-19. Moderate cases included patients with pneumonia but no signs of severe respiratory distress. Severe COVID-19 was defined by criteria such as oxygen saturation of less than 93% on room air at sea level, a respiratory rate greater than 30 breaths per minute, significant lung infiltrates greater than 50% within 24 to 48 h, or clinical signs of pneumonia with a high respiratory rate or oxygen requirement. Critical cases were marked by respiratory failure requiring mechanical ventilation, the presence of septic shock, or other organ dysfunction that necessitates intensive care management. 

### 2.4. Statistical Analysis

A sample size calculation was performed to ensure adequate power for detecting significant differences in cytokine levels between severe and nonsevere COVID-19 cases. Based on effect sizes derived from preliminary data and assuming a high effect size due to the expected pronounced differences in cytokine expression, we utilized an alpha of 0.05 and a power of 0.80. The calculation indicated that a minimum of 35 participants would be sufficient to discern significant effects. Data management and statistical analysis were conducted using SPSS version 26.0 (SPSS Inc., Chicago, IL, USA). The normality of data was tested using the Shapiro–Wilk test. Normally distributed variables were represented as means ± standard deviation (SD). Non-normally distributed variables were represented as median (IQR), while categorical variables were expressed as frequencies and percentages. Comparative analysis between groups (patients with severe vs. nonsevere COVID-19) involved Student’s *t*-test for continuous data and the Chi-square test for categorical variables. The patients were matched by age. To assess the predictive capability of laboratory parameters for COVID-19 severity, receiver operating characteristic (ROC) curves were generated. The area under the curve (AUC), along with sensitivity and specificity, was calculated to determine the best cutoff values of the parameters. The compound score 1 was calculated by summing the best cutoff values for CXCL, IL-1β, IL-6, IL-10, IL-17A, and MCP. The compound score 2 was calculated by a weighted sum to prioritize markers with higher individual AUC scores, aiming to enhance predictive accuracy by giving more influence to parameters that are more indicative of severity (CXCL, IL-1β, IL-10, and IL-17A received a weight of 12.5% of the total sum, while IL-6 and MCP received 25% of the total sum). Multivariate logistic regression was used to identify significant predictors of COVID-19 severity and calculate odds ratios. A *p*-value of less than 0.05 was considered statistically significant.

## 3. Results

The analysis of demographics and background characteristics of COVID-19 patients revealed no significant differences in age, gender, place of origin, vaccination status, or smoking habits between patients with severe and nonsevere disease outcomes. Specifically, the mean ages of patients with severe (71.9 years) and nonsevere COVID-19 (65.2 years) did not significantly influence disease severity. However, a notable exception was found in the oxygen saturation levels; patients with severe COVID-19 exhibited significantly lower mean oxygen saturation (80.9) compared with their nonsevere counterparts (96.2), with a highly significant *p*-value of less than 0.001 (Table 1).

Laboratory parameters measured at admission demonstrated significant differences between patients with severe and nonsevere COVID-19 outcomes. Notably, the median white blood cell count (WBC) was markedly lower in patients with severe COVID-19 (1.45 × 10^3^/L) compared with those with nonsevere conditions (9.10 × 10^3^/L). Additionally, other critical biomarkers such as C-Reactive Protein (CRP), lactate dehydrogenase (LDH), aspartate aminotransferase (AST), and alanine aminotransferase (ALT) also showed significant elevations in severe cases. Furthermore, fibrinogen levels were significantly higher in severe COVID-19 patients (662.00 mg/dL) compared with nonsevere patients (475.07 mg/dL), with a *p*-value of 0.002. The elevation of D-dimers in severe cases (1.82 μg/mL vs. 0.91 μg/mL, *p* = 0.035) further supported this finding (Table 2).

In the analysis of inflammatory markers between severe and nonsevere COVID-19 patients, significant variations were observed. Specifically, CXCL levels were markedly lower in nonsevere cases (298.07 pg/mL) compared with severe cases (408.13 pg/mL, *p* < 0.001). Similarly, IL-6 and MCP levels were significantly reduced in nonsevere COVID-19 patients, with mean values of 11.87 pg/mL and 240.67 pg/mL, respectively, compared with 30.35 pg/mL and 439.11 pg/mL in severe patients. Moreover, other inflammatory markers such as IL-10 and IL-1β also demonstrated higher mean concentrations in severe COVID-19 patients (13.45 pg/mL and 14.78 pg/mL) compared with those with nonsevere symptoms (6.37 pg/mL and 6.91 pg/mL, respectively), with IL-10 showing a statistically significant reduction (*p* = 0.006), as presented in Table 3.

IL-6 and MCP were the most promising markers, with IL-6 showing an optimal cutoff value of 18.74 while having the highest area under the curve (AUC) of 0.672. Similarly, MCP exhibited a cutoff of 335.68, with a sensitivity of 86.67% and specificity of 76.67%, resulting in an AUC of 0.629 and also displaying statistical significance (*p* = 0.001). Conversely, other inflammatory markers like NLR, CXCL, IL-1β, IL-10, and IL-17A demonstrated a lower diagnostic efficiency. 

**Figure 1:**
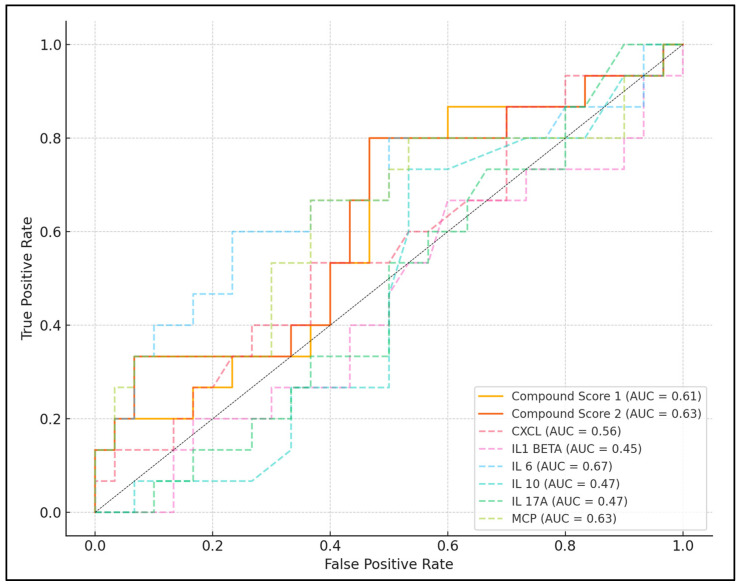
ROC curve analysis for severe COVID-19 predictors based on inflammatory scores; CXCL—chemokine Ligand; IL—interleukin; MCP—monocyte chemotactic protein; AUC—area under curve.

Furthermore, this study introduced compound scores, designed to enhance the predictive accuracy for severe COVID-19. Compound Score 1, established at a cutoff of 549.71, demonstrated a sensitivity of 80.00% and a specificity of 53.33%, with an AUC of 0.609 and a statistically significant *p*-value of 0.006. Compound Score 2, with a higher cutoff value of 768.30, showed improved results with a sensitivity of 86.67% and specificity of 63.33%. It achieved an AUC of 0.627 and a notable *p*-value of less than 0.001, which emphasizes its stronger predictive power compared with the first compound score (Table 4 and Figure 1). 

**Figure 2:**
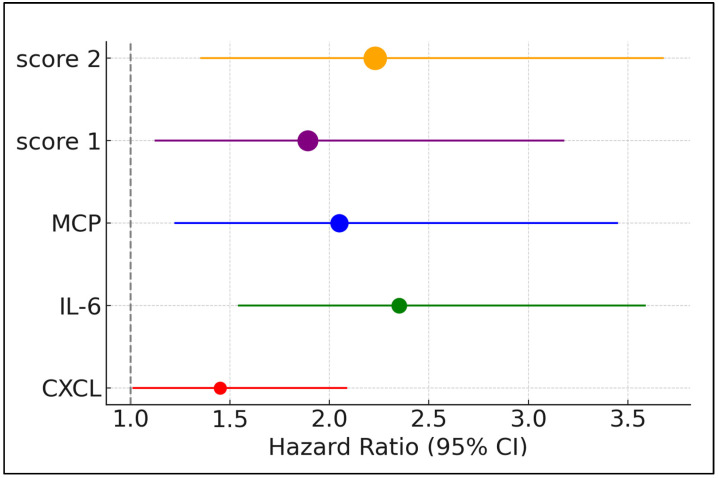
Forest plot of severe COVID-19 predictors based on inflammatory scores.

Among the markers, IL-6 and MCP exhibited statistically significant associations with severe COVID-19 outcomes. IL-6 showed a hazard ratio of 2.35, with a 95% confidence interval ranging from 1.54 to 3.59, and a *p*-value of 0.014. Similarly, MCP presented an HR of 2.05, with a 95% CI from 1.22 to 3.45, and a significant *p*-value of 0.007. Additionally, the compound scores, which integrate multiple inflammatory markers, also showed significant predictive value. Compound Score 2 had an HR of 2.23 (95% CI: 1.35–3.68) with a *p*-value of 0.002, indicating a strong association with severe COVID-19, higher than most single markers. Compound Score 1 similarly displayed significant predictive capability with an HR of 1.89 (95% CI: 1.12–3.18) and a *p*-value of 0.017. Conversely, markers such as NLR, CXCL, IL-1β, IL-10, and IL-17A did not show significant correlations (Table 5 and Figure 2).

## 4. Discussion

### 4.1. Literature Findings

The findings of this study clearly highlight the diagnostic potential of IL-6 and MCP as predictors of severe COVID-19, evidenced by their high hazard ratios and significant *p*-values. IL-6, with a hazard ratio of 2.35, indicates a more than twofold increase in the risk of severe disease outcomes associated with elevated levels. This is consistent with other studies that have identified IL-6 as a central mediator of the cytokine storm, often observed in severe cases of COVID-19, which leads to acute respiratory distress syndrome (ARDS) and other critical complications. Similarly, MCP, which showed a hazard ratio of 2.05, plays a crucial role in the recruitment of immune cells to infection sites, potentially exacerbating pulmonary inflammation and tissue damage.

Furthermore, the development and validation of compound scores as predictive tools in this study offer a novel approach to enhancing clinical assessments. Compound Score 2, in particular, demonstrated improved predictive accuracy with an HR of 2.23. This approach of integrating multiple inflammatory markers into a single predictive model aligns with the multifactorial nature of COVID-19, where the interplay between different cytokines and chemokines dictates the clinical trajectory. 

Other studies, although focused on similar goals of identifying laboratory biomarkers for severe COVID-19 outcomes, presented distinct findings given their different patient demographics and methods. One research work from Ethiopia [21] highlighted the neutrophil-to-lymphocyte ratio (NLR) with an adjusted relative risk (ARR) of 4.769 and sodium levels (ARR = 1.321) as significant predictors of COVID-19 severity among 429 patients. On the other hand, one study from Eastern India [22], analyzing a larger cohort of 7395 patients, developed a COVID-19 biochemical severity score, using parameters like high-sensitivity C-Reactive Protein (hs-CRP), lactate dehydrogenase (LDH), and interleukin-6 (IL-6), which showed a high odds ratio of 5.925 for predicting ICU admissions. 

Hariyanto et al. [23] found that elevated procalcitonin, CRP, D-dimer, and lactate dehydrogenase (LDH), alongside decreased albumin, were significant in predicting severe COVID-19 outcomes. Their meta-analysis, involving 4848 patients across 23 studies, showed mean differences in procalcitonin of 0.07 ng/mL, CRP of 36.88 mg/L, D-dimer of 0.43 μ/L, LDH of 102.79 U/L, and albumin decreased by 4.58 g/L. In contrast, Zhang et al. [24] investigated 289 hospitalized COVID-19 patients and identified elder age, underlying comorbidities, and specific laboratory markers such as leukocyte and neutrophil counts, NLR, CRP, procalcitonin, and D-dimer as key factors associated with severity and mortality.

Park et al. [25] investigated the prognostic utility of procalcitonin (PCT), presepsin, and the Veterans Health Administration COVID-19 (VACO) index in predicting 30-day mortality among 54 hospitalized COVID-19 patients. Their findings highlighted optimal cut-off values of 0.138 ng/mL for PCT and 717 pg/mL for presepsin with remarkably high hazard ratios of 15.9 for PCT and 26.3 for presepsin, suggesting strong predictive power for mortality. They concluded that combining these biomarkers with the VACO index significantly improved mortality predictions. In contrast, Kattner et al. [26] focused on the role of serum Krebs von den Lungen-6 (KL-6) in predicting outcomes in 157 patients with SARS-CoV-2 pneumonia, finding that a cut-off of 335 U/mL was a significant predictor of severe disease outcomes. 

d’Alessandro et al. [27] and Letellier et al. [28] both investigated the prognostic utility of Krebs von den Lungen-6 (KL-6) in COVID-19 patients, focusing on its ability to differentiate between severity levels and predict outcomes, respectively. In d’Alessandro’s study, KL-6 concentrations measured via two different methods (CLEIA and FEIA) significantly differentiated severe from nonsevere COVID-19 cases, with the Lumipulse G600II showing an AUC of 99.8% and a cut-off value of 448 U/mL, while the AIA360 demonstrated an AUC of 97.4% and a cut-off value of 398 U/mL. In contrast, Letellier’s study, which utilized a chemiluminescence enzyme immunoassay, found that higher KL-6 concentrations were significantly associated with in-hospital mortality (hazard ratio: 8.66) and worsened radiological outcomes. 

Recent studies have highlighted the critical role of cytokines in the progression and severity of COVID-19, providing potential biomarkers for predicting outcomes and guiding therapeutic interventions. Cabaro et al. analyzed cytokine patterns across two pandemic waves, revealing that cytokines such as IL-6 and IL-8 serve as robust predictors of disease severity and patient classification using machine learning techniques like LDA and CART [29]. Concurrently, Mandel et al. demonstrated that elevated levels of IL6 and TNFα are significant predictors of mortality, suggesting their role in identifying patients at higher risk of death within 30 days of hospital admission [30]. In the study by Basheer et al., a diverse array of cytokines, including TGF-β and IL-10, were linked to both mortality and severe lung involvement in COVID-19 patients, underscoring the complex interplay between inflammatory responses and clinical outcomes [31]. Herr et al. identified a suite of biomarkers, including IL-1ra and IL-8, through UMAP analysis, which effectively differentiated between patient outcomes and could facilitate more tailored treatment approaches [32].

Ochoa-Ramirez et al. focused on chemokines like MIG, MCP-1, and IP-10, which were found to be higher in patients with severe disease and could predict worse outcomes, indicating potential targets for therapeutic intervention [33]. Lastly, the study by Smail et al. pinpointed IL-10, IL-23, and TNF-α as excellent predictors of in-hospital mortality, highlighting their importance in the clinical management of COVID-19 patients [34].

### 4.2. Limitations

The sample size, although adequate for initial analyses, limits the generalizability of the results to larger, more diverse populations. Additionally, this study’s focus on hospitalized patients may introduce selection bias, as it does not consider asymptomatic or mildly symptomatic cases that do not require hospitalization. Furthermore, the exclusion criteria may have eliminated patients with co-infections that could provide insights into the compounded effects of multiple pathogens on cytokine levels. Using ELISA is also a limitation of the current study, considering other methods that are more accurate to detect and quantify cytokines Future studies should aim to include a broader spectrum of COVID-19 patients and possibly longitudinal sampling to assess cytokine levels over time.

## 5. Conclusions

This study confirms the significant predictive value of IL-6 and MCP for severe COVID-19, advocating for their inclusion in prognostic assessments of hospitalized patients. The utility of compound scores, particularly a weighted sum of IL-6, MCP, CXCL, IL-6, IL10, IL-17A, and IL-1β, were particularly stronger in predicting disease severity, reinforcing the potential of a multifaceted biomarker approach in managing COVID-19. By integrating these findings into clinical practice, healthcare providers can improve risk stratification, timely intervention, and ultimately patient outcomes in the face of this global health crisis. Further research to validate these findings in a broader population and over longer periods will be crucial in solidifying the role of these biomarkers in clinical settings.

## Figures and Tables

**Table 1:** Background characteristics and demographics of admitted patients with COVID-19.

Variables	Severe COVID-19 (*n* = 15)	Non-Severe COVID-19 (*n* = 31)	*p*-Value
Age (mean ± SD)	71.9 ± 8.8	65.2 ± 13.6	0.744
Age category			0.051
<40 years	0 (0%)	2 (6.5%)	
40–59 years	1 (6.7%)	11 (35.5%)	
≥60 years	14 (93.3%)	18 (58.1%)	
Gender			0.293
Men	7 (46.7%)	21 (68.8%)	
Women	8 (53.3%)	10 (31.3%)	
Place of origin			0.311
Rural	2 (13.3%)	10 (31.3%)	
Urban	13 (86.7%)	21 (68.8%)	
COVID-19 vaccination	Yes: 4 (26.7%)	Yes: 11 (34.4%)	0.793
Smoking			0.329
Yes	0 (0%)	4 (12.9%)	
No	9 (60.0%)	15 (48.4%)	
Past smoker	5 (33.3%)	9 (29.0%)	
Days since symptom onset (mean ± SD)	7.9 ± 5.6	4.9 ± 3.9	0.076
Days of hospitalization (mean ± SD)	11.1 ± 5.3	9.5 ± 6.6	0.782
Oxygen saturation (mean ± SD)	80.9 ± 7.7	96.2 ± 2.8	<0.001

SD—standard deviation.

**Table 2:** Common laboratory parameters measured at admission.

Variables	Normal Range	Severe COVID-19 (*n* = 15)	Nonsevere COVID-19 (*n* = 31)	*p*-Value
WBC (×10^3^/L)	4.0–10.0	1.45 (3.0)	9.10 (4.5)	<0.001
Hemoglobin (g/dL)	12.0–16.0	11.26 ± 1.74	12.45 ± 1.88	0.026
Neutrophils (×10^3^/L)	2.0–7.0	8.12 ± 3.91	6.74 ± 4.34	0.797
Lymphocytes (×10^3^/L)	1.0–3.0	2.30 ± 0.76	1.83 ± 0.87	0.020
Platelets (×10^3^/μL)	150–400	209.80 ± 110.12	262.94 ± 138.68	0.341
ESR (mm/h)	<20	58 (46)	51 (39)	0.286
Fibrinogen (mg/dL)	200–400	662.00 ± 140.76	475.07 ± 228.91	0.002
CRP (mg/L)	<5	51 (40)	18 (31)	<0.001
LDH	100–250	446 (213)	277 (209)	<0.001
AST (U/L)	0–40	57.00 ± 29.57	24.90 ± 6.51	<0.001
ALT (U/L)	0–40	48.07 ± 27.11	25.19 ± 16.24	0.004
Urea (mg/dL)	15–45	84 (61)	42 (33)	<0.001
Creatinine (mg/dL)	0.6–1.2	1.28 ± 0.86	1.09 ± 0.97	0.083
Blood glucose (mg/dL)	70–140	173.69 ± 102.21	126.03 ± 94.93	0.203
D-dimers (μg/mL)	0.0–0.5	1.82 ± 1.74	0.91 ± 1.58	0.035

AST—aspartate aminotransferase; ALT—alanine aminotransferase; CRP—C-reactive Protein; WBC—white blood cells; LDH—lactate dehydrogenase; ESR—erythrocyte sedimentation rate.

**Table 3:** Comparison of inflammatory markers and scores among COVID-19 patients.

Variables	Severe COVID-19 (*n* = 15)	Nonsevere COVID-19 (*n* = 31)	*p*-Value
NLR	8.22 ± 4.89	5.49 ± 6.75	0.057
CXCL	408.13 ± 83.52	298.07 ± 106.28	<0.001
IL-1β	14.78 ± 29.63	6.91 ± 4.81	0.152
IL-6	30.35 ± 17.01	11.87 ± 7.54	0.001
IL-10	13.45 ± 13.85	6.37 ± 1.11	0.006
IL-17A	16.42 ± 38.01	4.15 ± 8.93	0.092
MCP	439.11 ± 248.02	240.67 ± 112.36	0.004

NLR—neutrophil-to-lymphocyte ratio; CXCL—C-X-C motif chemokine ligand; IL-1β—interleukin-1 beta; IL-6—interleukin-6; IL-10—interleukin-10; IL-17A—interleukin-17A; MCP—monocyte chemoattractant protein.

**Table 4:** Best cutoff values for severe COVID-19 prediction.

Laboratory Parameter	Best Cutoff Value	Sensitivity	Specificity	AUC	*p*-Value
NLR	6.91	73.33%	61.29%	0.503	0.237
CXCL	342.04	66.67%	64.52%	0.564	0.191
IL-1β	8.80	50.00%	76.67%	0.448	0.583
IL-6	18.74	73.33%	86.67%	0.672	<0.001
IL-10	7.98	80.00%	33.33%	0.467	0.714
IL-17A	14.66	66.67%	63.33%	0.472	0.536
MCP	335.68	86.67%	76.67%	0.629	0.001
Compound score 1	549.71	80.00%	53.33%	0.609	0.006
Compound score 2	768.30	86.67%	63.33%	0.627	<0.001

NLR—neutrophil-to-lymphocyte ratio; CXCL—C-X-C motif chemokine ligand; IL-1β—interleukin-1 beta; IL-6—interleukin-6; IL-10—interleukin-10; IL-17A—interleukin-17A; MCP—monocyte chemoattractant protein.

**Table 5:** Regression analysis for severe COVID-19 development based on the inflammatory markers.

Factors above the Best Cutoff	Hazard Ratio	95% CI Lower	95% CI Upper	*p*-Value
NLR	1.23	0.85	1.78	0.299
CXCL	1.45	1.01	2.09	0.041
IL-1β	0.88	0.45	1.72	0.710
IL-6	2.35	1.54	3.59	0.014
IL-10	1.12	0.69	1.82	0.652
IL-17A	1.57	0.93	2.66	0.196
MCP	2.05	1.22	3.45	0.007
Compound score 1	1.89	1.12	3.18	0.017
Compound score 2	2.23	1.35	3.68	0.002

NLR—neutrophil-to-lymphocyte ratio; CXCL—C-X-C motif chemokine ligand; IL-1β—interleukin-1 beta; IL-6—interleukin-6; IL-10—interleukin-10; IL-17A—interleukin-17A; MCP—monocyte chemoattractant protein.

## Data Availability

Data are available on request from the authors.
